# Autophagy is Impaired in the Tibialis Anterior of Dystrophin Null Mice

**DOI:** 10.1371/currents.md.e1226cefa851a2f079bbc406c0a21e80

**Published:** 2013-11-22

**Authors:** Pietro Spitali, Paolo Grumati, Monika Hiller, Martina Chrisam, Annemieke Aartsma-Rus, Paolo Bonaldo

**Affiliations:** Department of Human Genetics, Leiden University Medical Center, Leiden, Netherlands; Department of Biomedical Sciences, University of Padova, Padova, Italy; Department of Human Genetics, Leiden University Medical Center, Leiden, Netherlands; Department of Molecular Medicine, University of Padova, Padova, Italy; Department of Human Genetics, Leiden University Medical Center, Leiden, Netherlands; Department of Biomedical Sciences, University of Padova, Padova, Italy

## Abstract

Background Duchenne muscular dystrophy is a lethal, progressive, muscle-wasting disease caused by mutations in the DMD gene. Structural remodelling processes are responsible for muscle atrophy and replacement of myofibers by fibrotic and adipose tissues. Molecular interventions modulating catabolic pathways, such as the ubiquitin-proteasome and the autophagy-lysosome systems, are under development for Duchenne and other muscular dystrophies. The Akt signaling cascade is one of the main pathways involved in protein synthesis and autophagy repression and is known to be up-regulated in dystrophin null mdx mice. Results We report that autophagy is triggered by fasting in the tibialis anterior muscle of control mice but not in mdx mice. Mdx mice show persistent Akt activation upon fasting and failure to increase the expression of FoxO3 regulated autophagy and atrophy genes, such as Bnip3 and Atrogin1. We also provide evidence that autophagy is differentially regulated in mdx tibialis anterior and diaphragm muscles. Conclusions Our data support the concept that autophagy is impaired in the tibialis anterior muscle of mdx mice and that the regulation of autophagy is muscle type dependent. Differences between muscle groups should be considered during the pre-clinical development of therapeutic strategies addressing muscle metabolism.

## Background

Duchenne Muscular Dystrophy (DMD) is the most common neuromuscular disorder. DMD is caused by the complete absence of the dystrophin protein, which leads to extensive muscle degeneration and regeneration followed by substitution of muscle with fibrotic and adipose tissues 1 ^,^ 2. No cure is yet available, but several therapeutic approaches aiming at reversal of the ongoing degeneration have been investigated in preclinical and clinical settings with disappointing results 3 ^,^ 4 ^,^ 5. Currently, drugs intended to induce skeletal muscle hypertrophy via Akt-mediated protein synthesis are in preclinical (e.g. valproic acid) or clinical (e.g. IGF-1) development 6 ^,^ 7 ^,^ 8 (see also http://clinicaltrials.gov/ct2/show/NCT01207908). IGF-1 is able to trigger Akt phosphorylation via class I PI3K 9 ^,^ 10 , which in turn induces a series of biochemical changes leading to protein synthesis via the mammalian target of rapamycin (mTOR) pathway 11 ^,^ 12 ^,^ 13 . At the same time, Akt is able to repress catabolic pathways, such as macroautophagy (hereafter referred as autophagy) and ubiquitin-proteasome, leading to muscle atrophy 14 ^,^ 15 . This repression can occur in a transcriptional and non-transcriptional fashion. Indeed, it is known that mTOR inhibition by rapamycin induces autophagy without affecting gene expression, while Akt can repress the transcription factor FoxO3. This transcription factor is involved in the transcriptional activation of the atrophy genes *Atrogin1* and *MuRF1* and the autophagy gene *Bnip3* 16 ^,^ 17 . Therefore, the autophagy-lysosome system might also be a potential target for therapeutic intervention for muscular dystrophies.

The autophagic pathway is responsible for the removal of unfolded/toxic proteins as well as dysfunctional/abnormal organelles. It is constantly active in skeletal muscle and is involved in several conditions such as denervation, cachexia and fasting 18 . We recently reported that autophagy is impaired in collagen VI-related myopathies and that induction of autophagy can rescue myofibers defects of collagen VI deficient mice 19 ^,^ 20 . The Akt/mTOR axis is one of the key pathways regulating autophagy. Previous studies showed that Akt signaling is affected in dystrophin null *mdx* mice 21 . In fact, Akt signaling is strongly enhanced in 4-week-old *mdx* mice, *i.e.* the period when extensive muscle regeneration is occurring 22 . This enhancement of Akt signaling decreases over time with a slight up-regulation in 3-month-old mice 23 ^,^ 24 and hardly any up-regulation in older mice 25 . Recently it was reported that both activation of autophagy by an AMPK agonist and inhibition of autophagy by Akt activation via valproic acid could ameliorate the dystrophic phenotype of *mdx* mice 6 ^,^ 26 . Furthermore, it was shown that autophagy is impaired in both glycolytic and oxidative muscles of *mdx* mice 27 . In the present study, we analyzed the Akt/mTOR pathway under basal conditions and after fasting in *mdx* and wild-type mice. We found a persistent activation of the Akt/mTOR pathway after fasting in the *mdx* mice tibialis anterior but not in the diaphragm. Taken together, these data show that abnormal Akt signaling differentially impacts the regulation of the autophagy machinery in diverse dystrophin deficient muscles.

## Methods

**Ethical approval**

All procedures were approved by the Animal Welfare Commission of the Leiden University Medical Center (work protocol 11071). The institution is authorized by the government to judge the proposals according to the law. All experiments were performed in accordance with the regulations for animal experimentation.

**Mice**

C57BL/10ScSn-mdx/J (*mdx*) and control C57BL/10 mice were fed ad libitum with chow until 16 weeks of age. At this age, mice were divided into the fed or fasting groups (4-5 mice per group). Fasting started at 9 am in the morning and lasted for 24 hours. Mice from both groups were then sacrificed by cervical dislocation. Tibialis anterior and diaphragm muscles were harvested and immediately frozen in liquid nitrogen before further processing.

**q-RT-PCR analysis**

Total RNA was isolated using Tripure reagent as described previously 28 . The RNA concentration was measured on a Nanodrop (Nanodrop Technologies, USA) and integrity was checked with a total RNA nano chip assay on the Agilent 2100 bioanalyzer (Agilent, the Netherlands). cDNA synthesis was performed using random hexamer primers and gene expression levels were determined by Sybr Green based Real Time qPCR on the Roche Lightcycler 480 (Roche Diagnostics Ltd, UK). All primer pairs used spanned at least one splice junction to avoid contamination with genomic DNA amplification. Relative expression was determined using Gapdh as reference gene, while primer efficiencies were determined with LinReg PCR version 11.3.

**Western Blot**

Frozen muscles were homogenized by grinding in liquid nitrogen, lysed and immunoblotted as previously described 19 . When needed, membranes were stripped using a stripping buffer (25 mM glycine, 1% SDS, pH 2.0) and reprobed. The following antibodies from Cell Signalling Technologies were used: rabbit polyclonal anti-Akt; rabbit monoclonal (clone 193H12) anti-phospho-Akt (Ser473); rabbit polyclonal anti-4EBP1; rabbit polyclonal anti-phospho- 4EBP1 (Ser65). The rabbit polyclonal anti-LC3B was from Tema Ricerca and mouse monoclonal anti-GAPDH was from Millipore. Western blots were performed for a minimum of three independent experiments. Densitometric quantification was carried out using ImageJ software.

**Statistical Analysis**

To test whether changes in gene expression levels were significant between fed and fasted mice, we used one-way ANOVA followed by Post-hoc tests using the Bonferroni correction for multiple testing. *P*-values lower than 0.05 were considered significant.

## Results

**Autophagy is impaired in *mdx* mice**

To investigate autophagy regulation in *mdx* mice, we chose the tibialis anterior and diaphragm muscles as they are examples of glycolytic and oxidative muscles, respectively 19 ^,^ 27 ^,^ 41 . We investigated 16-weeks-old mice, since it is known that *mdx* mice undergo extensive muscle regeneration between 6 and 12 weeks of age, which could confound the results. Notably, this muscle regeneration does not occur in Duchenne patients 22 .

We first assessed Akt phosphorylation in wild-type and *mdx* muscle from mice that were fed ad libitum and did not observe significant differences between the two groups (Figure 1). In agreement with this, no differences were found in the phosphorylation state of the eukaryotic translation initiation factor 4E-binding protein 1 (4EBP1), which dissociates from the eukaryotic translation initiation factor 4E (eIF4E) and activates mRNA translation when phosphorylated. Furthermore, no differences were observed in the lipidated form of the microtubule-associated protein-1 light chain 3 (LC3-II), which is produced during the autophagosome formation 29 ^,^ 30 . Fasting for 24 hr induced autophagy in wild-type and *mdx* mice diaphragm, leading to decreased phosphorylation of Akt and 4EBP1 and increased levels of LC3-II. Conversely, 24 hr fasting induced autophagy in tibialis anterior muscle of wild-type but not of *mdx* mice. Indeed, only the tibialis anterior of wild-type mice showed autophagy induction, while in *mdx* mice Akt and 4EBP1 remained phosphorylated. The LC3-II form was also less abundant in the tibialis anterior of *mdx* mice, confirming that this muscle was resistant to autophagy induction (Figure 1).

**Autophagy is impaired in mdx mice:**
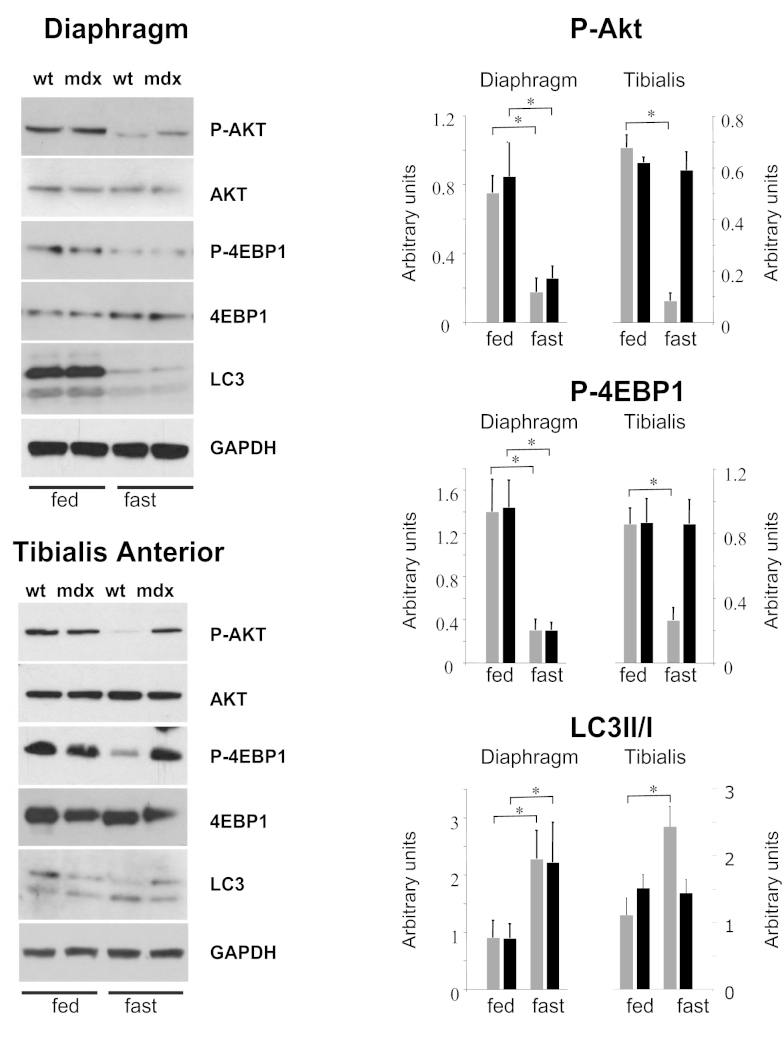
Western blot of phosphorylated and total Akt, phosphorylated and total 4EBP1, and LC3 lipidation in diaphragm and tibialis anterior muscles of wild-type and *mdx *mice at basal level and after 24 hour fasting (n=4-5). GAPDH was used as loading control. Densitometric quantification of phospho-Akt, phospho-4EBP1 and ratio between LC3-II vs LC3-I form are also shown (*P<0.05). Expression levels are represented as arbitrary units. Error bars indicate s.e.m.

**Autophagy impairment is mediated by FoxO3 transcription factor**

Akt is known to be one of the most potent modulators of autophagy and inhibition of the IGF-1/Akt pathway during fasting stimulates autophagy mainly via an mTOR independent mechanism 18 . Therefore, we studied the expression of some regulatory genes involved in autophagy induction, such as *Beclin1*. Furthermore, we focused in particular on FoxO3-regulated genes, such as *LC3* and *Bnip3*. The latter is the main gene involved in fasting-induced autophagosome formation in muscle 16 and a key regulator of the autophagic removal of mitochondria 31 ^,^ 32 . In the diaphragm of wt and *mdx* mice, fasting for 24 hours induced potent up-regulation of the autophagy activation genes *Bnip3* and *Beclin1* as well as the ubiquitin-ligase genes *Atrogin1* and *MuRF1* (Figure 2A). However, in the glycolytic tibialis anterior muscle, fasting induced *Bnip3* expression in wild-type mice only, while no difference was observed between fed and fasted *mdx* mice. Similar results were obtained for *Atrogin1*, an atrophy-related ubiquitin ligase also regulated by FoxO3.The levels of *MuRF1*, another atrophy-related ubiquitin ligase regulated by NF-κB, were increased in the muscles of fasted *mdx* and wild-type mice compared to fed mice. No significant changes were observed in the expression of *LC3 *and *Beclin1* (Figure 2B).

**Autophagy impairment is mediated by FoxO3 transcription factor.**
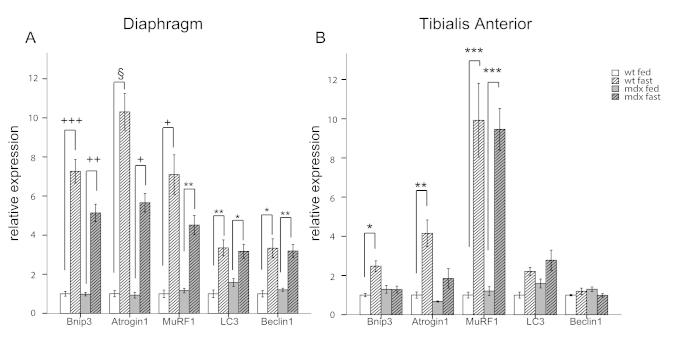
q-RT-PCR analysis showing the quantification of the genes involved in the autophagy regulation. *Bnip3*, *Atrogin1*, *MuRF1*, *LC3* and *Beclin1* are shown for diaphragm (A) and tibialis anterior (B) muscles (n=4-5). White bars represent fed wild-type mice, hatched white bars represent fasted wt mice, grey bars represent fed *mdx* mice and hatched grey bars represent fasted *mdx* mice. (* *P*<10^-2^, ** *P*<10^-3^, *** *P*<10^-4^, ^+^ *P*<10^-5^, ++ *P*<10^-6^, +++ *P*<10^-7^, § *P*<10^-8^). Bars represent the mean expression relative to the wt fed mice group which is set to 1. Error bars indicate s.e.m.

## Discussion

DMD is the most severe form of muscular dystrophy and also the most common neuromuscular disorder. Transcriptomic and proteomic studies have reported major metabolic and physiological changes in DMD patients and animal models, including mitochondrial defects 22 ^,^ 33 ^,^ 34 ^,^ 35 ^,^ 36 . Structural remodelling processes, such as extensive muscle regeneration, can compensate for dystrophin absence during the early stages of the disease in which young patients are still able to walk. To identify potential therapeutic targets for DMD, several studies focused on pathways involved in muscle hypertrophy, such as myostatin inhibition or IGF-1/Akt activation 6 ^,^ 37 . Akt stimulates protein synthesis and hypertrophy by inhibiting the tuberous sclerosis complex (TSC), which inhibits mTOR 38 . At the same time, Akt inhibits autophagy by phosphorylating the FoxO3 transcription factor 16 ^,^ 39 . Several reports have shown that Akt is more active in *mdx* compared to wild-type mice, although differences in Akt signaling were reported to be age- and muscle-dependent. Indeed, it is known that *mdx* mice at about 6-12 weeks of age show extensive muscle regeneration when compared to older *mdx* mice 22 and that Akt signaling decreases over time in *mdx* mice 25 .

We recently reported defective autophagy regulation in another animal model of muscular dystrophy, the collagen VI null (*Col6a1* ^–/–^) mouse. In *Col6a1* ^–/–^ mice, autophagy is strongly impaired in the tibialis anterior muscle, while in the diaphragm the autophagy machinery is less compromised 19 . Here we show comparable results in the *mdx *mouse, where the autophagy pathway is normally regulated in the diaphragm and impaired in a highly glycolytic muscle such as the tibialis anterior.

Autophagy impairment in *mdx* tibialis anterior muscle could be due to persistent Akt activation; this however remains to be tested by e.g. knocking down Akt during the period of food deprivation. Differential physical activity levels between *mdx* and wt mice could also account for this difference, since it has been shown that exercise can influence autophagy 42 . However mice involved in our experiment were not exercised, even though it is known that *mdx* mice move less compared to wt mice. Future experiments will also need to determine what causes the different response to fasting observed in *mdx* muscles. We hypothesize that muscle condition could be a factor, since the diaphragm is the most severely affected muscle in *mdx* mice while the tibialis anterior is mildly affected; myofiber composition could also participate to the difference observed between the two muscles as tibialis anterior and diaphragm have been considered in the past as examples of glycolytic and oxidative muscles 41 .

These findings are partially in line with recently published data demonstrating that autophagy is equally impaired in both tibialis anterior and diaphragm muscles 27 . A possible reason for the difference between our experiment and the one by De Palma and colleagues is the fasting time which was 24 hours in our case compared to 15 hours in the article by De Palma et al. It is possible that 15 hours are not sufficient to trigger autophagy in *mdx* mice diaphragm. Our current data clearly shows a differential autophagy response in distinct muscle types and we think that this should be taken into account when designing therapeutic strategies targeting this pathway. Autophagy activation in the diaphragm of *mdx* mice was shown to be beneficial either via an AMPK agonist 26 that rescued the PTP function, or by rapamycin mediated inhibition of mTOR that decreased the number of necrotic and regenerating fibers 40 . On the contrary, the same treatment did not lead to mTOR inhibition in the tibialis muscle, underlining the differences between glycolytic and oxidative muscles. It is known that Akt overexpression in the glycolytic gastrocnemius of *mdx* mice is able to protect from isometric force drop after eccentric contractions *in vivo* 8 . The positive role of Akt signaling in glycolytic muscles is also confirmed in the plantaris muscle, where Akt signaling is induced, but represents a limiting factor to muscle remodeling following mechanical overloading 23 . However, it has also been reported that IGF-1 up-regulation in the oxidative *mdx* diaphragm can cause hypertrophy and hyperplasia reducing fibrosis 24 .

Our data demonstrate that autophagy is differentially regulated in tibialis anterior and diaphragm muscles of *mdx *mice. Given the difference in fiber type composition between human and mouse (humans do not have the glycolytic type IIB myosin heavy chain 43 ), the results obtained in mice diaphragm better represents the fiber type composition in human skeletal muscle. This suggests that both AMPK agonists and IGF-1 could be good candidates to test in patients given the positive results obtained in *mdx *mice, even though the two approaches aim at diametrically opposite biochemical results. Before clinical experimentation, however, therapeutic interventions aiming to interfere with the Akt autophagy pathway should be carefully evaluated considering the differences between muscle groups and preferably show that both muscle types respond positively to the treatment.

## Conclusions

This study demonstrates that autophagy was not induced after fasting in the tibialis anterior muscle of dystrophin null mice. Autophagy was potently induced in the diaphragm muscle of *mdx *as well as wt mice. The difference between the two types of skeletal muscle underlines the fact that a specific treatment to improve muscle condition could have a different effect in different types of muscle.

## Corrisponding authors

**Annemieke Aartsma-Rus**

Human Genetics Department

Leiden University Medical Center

A.M.Aartsma-Rus@lumc.nl

Paolo Bonaldo

Department of Molecular Medicine

University of Padova

bonaldo@bio.unipd.it

## Keywords

Duchenne Muscular Dystrophy; Autophagy; Macroautophagy; *mdx* mouse.

## Competing Interests

The authors have declared that no competing interests exist.
